# UV Light–Induced Aggregation of Titania Submicron Particles

**DOI:** 10.3390/mi7110203

**Published:** 2016-11-08

**Authors:** Can Zhou, Yashar Bashirzadeh, Timothy A. Bernadowski, Xiaoyu Zhang

**Affiliations:** Department of Mechanical & Aerospace Engineering, Old Dominion University, Norfolk, VA 23529, USA; czhou001@odu.edu (C.Z.); ybash001@odu.edu (Y.B.); tbern008@odu.edu (T.A.B.J.)

**Keywords:** Derjaguin–Landau–Verwey–Overbeek (DLVO), Zeta potential, rutile, anatase, electrokinetics, mobility

## Abstract

In this study, aggregation of TiO_2_ (rutile and anatase) submicron particles in deionized (DI) water under ultra-violet (UV) light irradiation was investigated. While no aggregation was observed in the dark, rutile and anatase submicron particles started aggregating upon application of UV light and ceased aggregation in about 2 and 8.4 h, respectively. It has been demonstrated that UV light directly mitigated the particle mobility of TiO_2_, resulting in a neutralization effect of the Zeta potential. It was also observed that rutile particles aggregated much faster than anatase particles under UV radiation, indicating that the Zeta potential of as-prepared rutile is less than that of anatase in deionized (DI) water. In addition, the interaction energy of rutile and anatase particles was simulated using the Derjaguin–Landau–Verwey–Overbeek (DLVO) model. The results showed a significant reduction of barrier energy from 118.2 k_B_T to 33.6 k_B_T for rutile and from 333.5 k_B_T to 46.1 k_B_T for anatase, respectively, which further validated the remarkable influence of UV irradiation on the aggregation kinetics of rutile and anatase submicron particles. This work presents a further understanding of the aggregation mechanism of light-controlled submicron particles and has a promising potential application in environmental remediation.

## 1. Introduction

Aqueous micromotors driven by external energy from either a chemical reaction with an electrolyte or an environmental stimulus such as light, magnetic fields, temperature gradients, concentration gradients, etc., have drawn a lot of research interest [1,2,3,4]. Due to their small size and simple manipulation, micromotors composed of micro- or nanoparticles have promising applications in drug delivery, cell separation, bacterial degradation, and chemical decomposition [5,6,7]. Titania (TiO_2_) particles have been commonly used in these micromotors. As an *n*-type semiconductor with strong photocatalytic activities, titania has been widely investigated for photoelectrochemical water splitting to produce hydrogen [8], following the work of technological pioneers Fujishima and Honda [9].

Light-induced behaviors of titania particles have become more researched over recent years. Anatase TiO_2_ particles (0.2–2.5 µm) were reported to exhibit ultra-violet (UV)-induced microfireworks with tracer particles such as SiO_2_ (2.34 µm) and amidine polystyrene (2.5 µm) [10]. Despite oppositely charged properties, both tracer particles would be repelled by TiO_2_ particles in the dark whereas they would be attracted towards TiO_2_ particles upon UV light exposure. According to the proposed diffusiophoretic model, the repeatable light switch effect was believed to be caused by the concentration gradient of photogenerated chemical species, osmotic propulsion, and surface charge interaction [10]. Photo-induced disaggregation of TiO_2_ nanoparticles (27 ± 4 nm) was observed by Bennett et al. [11] and Zhou et al. [12]. It was reported that TiO_2_ nanoparticles would disaggregate from core clusters upon light irradiation and re-aggregate in the dark due to Brownian motion. Furthermore, infrared light was found to contribute to disaggregation of nanoparticles due to thermal energy input and induced vibration. The decrease of the Zeta potential under UV irradiation was insufficient to affect the balance of Derjaguin–Landau–Verwey–Overbeek (DLVO) forces. However, Sun et al. reported an opposite phenomenon in which UV light could induce aggregation of TiO_2_ nanoparticles (21 nm) [13]. The results showed that the hydrodynamic size increased from 252 nm to 623 nm after 50 h of UV irradiation. It was explained that due to the formation of hydroxyl groups under UV irradiation, the surface charges of TiO_2_ nanoparticles were reduced over time. It was concluded that the decrease of the repulsive force between nanoparticles facilitated TiO_2_ aggregation. In addition to those investigations on pure TiO_2_ particles, TiO_2_-graphene (TiO_2_: 70–80 nm) composite nanoparticles were reported to aggregate after 20 h of UV irradiation due to a decrease in hydrophilicity [14,15]. Those aforementioned studies mainly focused on the behaviors of TiO_2_ particles induced by light at a nano-scale. As the particle size increases, TiO_2_ may exhibit different photocatalytic properties and electrokinetic behaviors. To our best knowledge, the study of UV light–induced movements of micron or submicron TiO_2_ particles is limited.

In this work, UV light–induced aggregation of submicron particles in aqueous suspensions was demonstrated using two different polymorphs of TiO_2_, rutile and anatase. The objective was to investigate the factors that affect the particle aggregation, including irradiation duration, particle mobility, Zeta potential, and polymorphism. In addition, theoretical models were implemented to correlate and explain the experimental results.

## 2. Experiment

### 2.1. Aggregation Experiments

The aggregation experiments were conducted in an enclosed reservoir made of polydimethylsiloxane (PDMS) on a glass slide. The reservoir’s dimensions are 40 mm × 10 mm × 2 mm. The suspensions of TiO_2_ were injected into the reservoir and then sealed to prevent air intrusion. A 200 W solar simulator (Newport Corp., Irvine, CA, USA) with a Xe-Hg acr lamp was used as a light source. UV light was actuated using a dichroic mirror (Model 66217, Newport Corp.) that primarily reflects 260–320 nm light generated by the solar simulator. The experimental observations were performed via an optical microscope (Nikon TE2000-U, Nikon Instruments, Tokyo, Japan).

As-prepared rutile (500 nm) and anatase (325 mesh) powders were obtained from US-NANO and Sigma-Aldrich (St. Louis, MO, USA), respectively. It has been verified under the microscope that the particle size of anatase powder from Sigma-Aldrich is submicron. For preparation of TiO_2_ suspension, rutile and anatase powders were directly added into deionized (DI) water, respectively, by a ratio of 3 mg:150 mL and the microsphere concentration was around 0.25 mM [16]. After manually stirring, an ultrasonic bath (VWR 501, Radnor, PA, USA) and/or magnetic stirring (Fisher Scientific Isotemp, Pittsburgh, PA, USA) at 1000 rpm was used to form suspensions and disaggregate large particles. Subsequently, the suspensions were injected into the prepared enclosed reservoir for experimentation. The actual particle sizes were around 1 μm for both rutile and anatase suspensions, possibly due to some difficulties encountered while breaking nano TiO_2_ agglomerates in the ultrasonic bath [17].

### 2.2. Particle Mobility Measurement

The particle mobility under UV light in DI water was measured in a microchannel made of PDMS only. The microchannel consisted of a straight cylindrical conduit (Inner Diameter, ID = 0.3 mm) connected with two cubic reservoirs at both ends. An electric field was applied through two silver electrodes embedded outside the microchannel using a Direct current (DC) power supply (Circuit Specialists CSI20002S, Tempe, AZ, USA). Particle movement and velocity within the microchannel due to the electric field was visually recorded by the optical microscope. Lastly, the mobility was calculated by dividing the velocity of the particles by the strength of the electric field.

## 3. Theory

### 3.1. Particle Mobility and Zeta Potential

Zeta potential is an electrokinetic term that has been widely used to interpret the agglomeration and disaggregation of colloidal dispersions [18]. The Zeta potential represents the net electrical charge of a confined region. It is believed that when the Zeta potential becomes neutral, particles become more attractive so that they tend to agglomerate [13]. The aggregation of TiO_2_ particles in the present research can be interpreted by using the Zeta potential if it incrementally becomes more neutral under UV irradiation.

Direct measurement of the Zeta potential was not a viable option in our lab; therefore, an indirect method, measuring the particle mobility, was adopted. The particle mobility has a direct relationship with the Zeta potential, as shown in Equation (1) below. The particle mobility (*U*) is defined as the ratio of the drift velocity (*v_d_*) divided by the electric field (*E_z_*): *U* = *v_d_*/*E_z_*. Note that practically, in these experiments, the PDMS microchannel is usually negatively charged in solution over a wide range of pH [19]. Therefore, an electrical double layer containing net cations forms near the PDMS/electrolyte interface, leaving net mobile anions inside the microchannel. Electroosmotic flows from anode to cathode are generated in the center of the microchannel when an electric field is applied. Such fluid motion affects the movement of TiO_2_ particles. Hence, the measured mobility was actually the sum of the electrostatic mobility and electroosmotic flow: *U*_m_ = $U_{{\text{TiO}}_{2}}$ + *U*_os_. The Zeta potential of particles can be linearly correlated with electrostatic mobility based on Henry’s equation: [20,21]

(1) $$\zeta =\frac{2\mu U_{{\text{TiO}}_{2}}}{3\varepsilon_{r}\varepsilon_{0}}f\left(\kappa r\right)$$

(2) $$f\left(\kappa r\right)=1+\frac{1}{2}{\left[1+\left(\frac{2.5}{\kappa r\left[1+2\text{exp}\left(-\kappa r\right)\right]}\right)\right]}^{-3}$$

where μ is the solution viscosity; ε*_r_* and ε_0_ are the relative dielectric constant and the vacuum electrical permittivity respectively; *r* is the particle diameter; κ is the inverse Debye length in which κ = 2.32 × 10^9^(∑*C_i_Z_i_*^2^)^0.5^, where *C_i_* and *Z_i_* are the concentration and valency value of ion *i*; *f*(κ*r*) is Henry’s function and varies from 1.0 to 1.5, depending on different situations.

### 3.2. DLVO Model

Another viable theory to interpret the aggregation of aqueous TiO_2_ suspensions is the classical DLVO model. The DLVO model integrates the counteractions of the van der Waals attraction energy (*V*_VDW_) and the electrical double layer (EDL) repulsion energy (*V*_EDL_) among particles, which can be expressed using the following equations [16]:

(3) $$V_{\text{VDW}}=-\frac{A_{131}}{6}\left[\frac{2r^{2}}{d\left(4r+d\right)}+\frac{2r^{2}}{{\left(2r+d\right)}^{2}}+\text{ln}\frac{d\left(4r+d\right)}{{\left(2r+d\right)}^{2}}\right]$$

(4) $$V_{\text{EDL}}=2\pi r\varepsilon_{r}\varepsilon_{0}\zeta^{2}\text{ln}\left[1+\text{exp}\left(-\kappa d\right)\right]$$

where *A*_131_ is the Hamaker constant for nanoparticle-water-nanoparticle (6 × 10^−20^ J) [13]; *d* is the distance between two particles; and *n*_∞_ is the number concentrations of bulk ions.

The stability of a suspension is usually characterized by the total interaction energy (*V*_T_), which is the summation of *V*_VDW_ and *V*_EDL_. In a stable suspension, the total interaction energy acts as an energy barrier that resists particle aggregation. It is hypothesized that UV light affects the total interaction energy and results in a lower *V*_EDL_ and barrier energy. Consequently, particles start to aggregate, making the suspension unstable [13]. The DLVO model is similar to the Zeta potential and was validated by the experimental results.

## 4. Results and Discussion

### 4.1. Aggregation under UV Light

**Figure 1:**
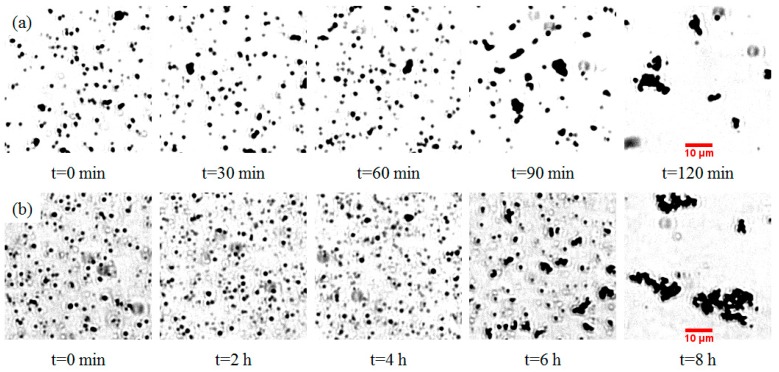
Microscopic images of ultra-violet (UV) light–induced aggregation of (**a**) rutile submicron particles over 120 min and (**b**) anatase submicron particles over 8 h in deionized (DI) water.

**Figure 2:**
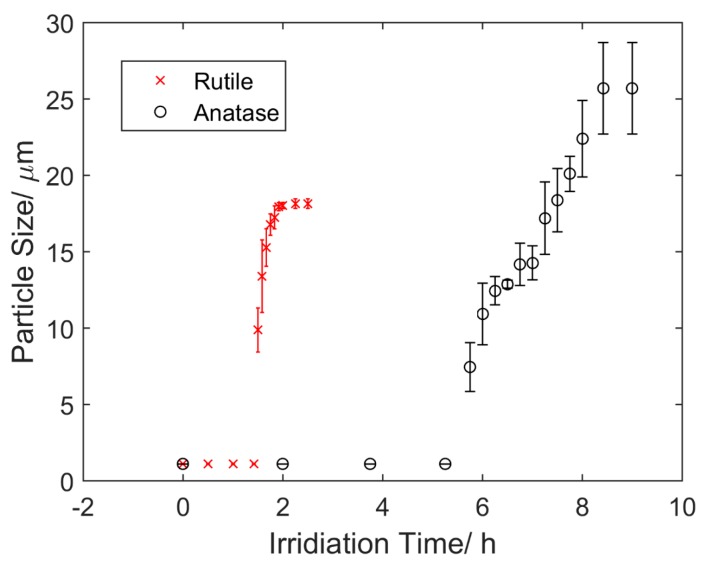
Size change of the rutile and anatase submicron particles in DI water as a function of irradiation time. The UV light was turned on at 0 h.

The aggregation of TiO_2_ submicron particles was investigated through visualization using an optical microscope. Prior to the application of UV light, both rutile and anatase suspensions were kept in a dark environment for 10 h to ensure minimum aggregation. Figure 1 shows the changes of rutile (Figure 1a) and anatase suspensions (Figure 1b) under UV light over different time periods. It was observed that the aggregation rate of rutile particles was much faster than that of anatase particles. A quantified version of aggregation that shows the changes of particle sizes over time is illustrated in Figure 2. Both rutile and anatase suspensions went through a slow start-up and then a rapid increase in terms of particle size during aggregation. It was illustrated that the rutile particles coagulated significantly faster starting from about 1.5 h, with an average growing rate of 1.23 µm/s in diameter, until their average sizes peaked at 2 h. After this time, no further aggregation was clearly observed and the ultimate average particle diameter remained around 18.15 µm. In comparison, the accumulation of anatase particles in the suspension showed a similar trend but in a much slower process. The rapid growth of anatase particles started at 5.75 h and plateaued at 8.4 h. During that period, the average growing rate was 0.22 µm/s in diameter and the ultimate average particle size was around 25.7 µm.

### 4.2. Particle Mobility

In order to further investigate the UV-induced aggregation electrokinetics, particle mobility tests were conducted using a lab-made microchannel. First, 20 V DC was applied through the electrodes which were spaced 20.2 mm apart. A regular mobility test was conducted under UV light with 5 min intervals, following a baseline test in the dark condition. The mobility tests lasted approximately 1 h, beyond which optical observations became difficult as sediments started blurring images in the microchannel. Particle movement in the microchannel is actually the net effect of the electroosmotic flow and electrostatic movement. The electroosmotic flow was assumed to remain stable throughout all the mobility tests. In addition, PDMS is highly transparent to UV light and its degradation under UV irradiation was assumed to be negligible.

**Figure 3:**
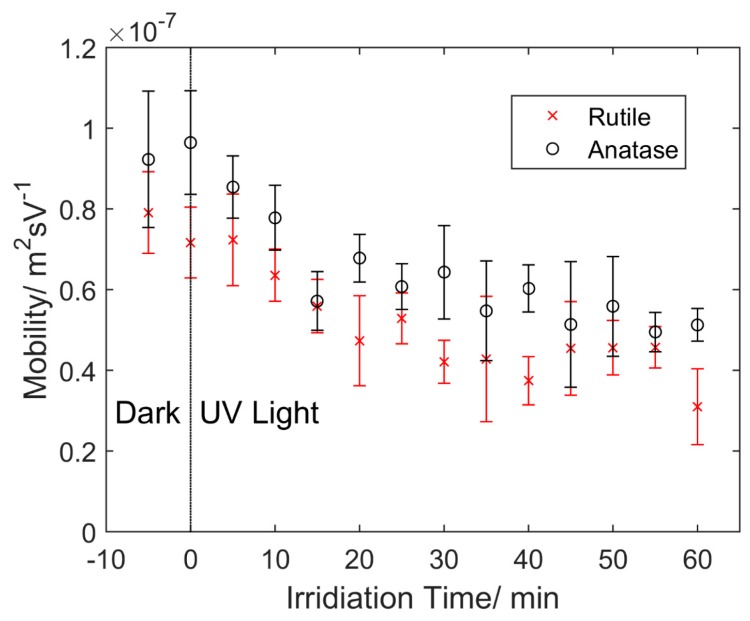
Measured particle mobility of the rutile and anatase submicron particles over time. The UV light was turned on at 0 h. The electric field strength was 0.99 V/mm.

The electrostatic movement of TiO_2_ particles due to the surface potential exhibited the same variance with the measured mobility depending on the irradiation time, as shown in Figure 3. The mobility of both rutile and anatase immediately decreased upon application of UV light. During the first 15 min, the effect was remarkable, with a decrease of 2.32 × 10^−8^ m^2^·s/V for rutile and 3.51 × 10^−8^ m^2^·s/V for anatase. Over 1 h of UV irradiation, the overall magnitude decrease of mobility for rutile and anatase was about 60.8% and 46.4%, respectively. Rutile exhibited a lower mobility than anatase with an average difference of 1.37 × 10^−8^ m^2^·s/V throughout the test. This corresponded to a lower Zeta potential of 12.1 mV based on Equations (1) and (2), and indicated a lower surface charge, and thus faster aggregation rate for rutile particles.

### 4.3. Discussion

UV light–induced aggregation was observed in both aqueous rutile and anatase suspensions that contained submicron particles. Such a phenomenon was attributed to the strongly photocatalytic activities of TiO_2_, which is an intrinsic *n*-type semiconductor with a wide bandgap (3.0–3.2 eV) [22]. Both rutile and anatase have been widely used as the photocatalysts for photoelectrochemical water splitting [8]. The wide bandgap of TiO_2_ determines the threshold of the light frequencies required for exciting electrons from the valence band to the conduction band. Therefore, a UV light source was required in those experiments.

The changes of the Zeta potential over time can be used to interpret UV light–induced aggregation of TiO_2_ particles observed in the experiments. Zeta potential has been used as an index of stability of colloidal dispersions [18]. The magnitude of the Zeta potential indicates the affinity between a particle and its adjacent ones. As the Zeta potential approaches neutral, the particles will have less resistance to aggregation, and vice versa. The Zeta potential of TiO_2_ particles is strongly relevant to the background pH value (or proton concentration) and the measured negative values in DI water (pH ≈ 6.5) [18]. Upon UV irradiation, it is believed that hydroxyl groups (·OH) form on the TiO_2_ particle surfaces, resulting in a higher local proton concentration (lower local pH). Consequently, the Zeta potential increases and becomes more neutral. This electrochemical process has been explained and verified by Fujishima et al. [23,24,25]. When illuminated by UV light, photogenerated electron-hole pairs generated on the TiO_2_ surfaces enable chemical redox reactions. Specifically, Ti^4+^ ions will combine with electrons and be reduced to Ti^3+^ ions, whereas the positive holes left will oxidize the bridging O atoms to produce oxygen vacancies in the lattice. Such vacancies then absorb water molecules and generate acidic bridging hydroxyls (pKa = 2.9) [13]. The chemically reactive hydroxyls are able to trap extra holes and subsequently dissociate water molecules. The final products are more concentrated protons, leading to the localized reduction of pH. Consequently, the Zeta potential of TiO_2_ particles becomes more and more neutral.

The remarkable difference of the time duration of the aggregation between rutile and anatase was observed repeatedly in the experiments. As illustrated in Figure 3, the as-received rutile particles show less mobility than anatase ones. This indicates the initial Zeta potential of rutile was more neutral than that of anatase. However, none of the existing theory can be used to explain such a phenomenon, other than the possible different processes of powder synthesis from individual vendors, as pointed out in References [26,27]. Kosmulski et al. reviewed the research on the point zero charge (PZC) of rutile and anatase and their summary indicated that the Zeta potential of TiO_2_ is not sensitive to its common crystal phases [26]. That conclusion basically excludes the cause due to different polymorphs of TiO_2_. In addition, the particle sizes of those rutile and anatase submicron particles used in the experiments outlined in this work were similar. Therefore, the sizing effect on Zeta potential [28] cannot be fully used to justify the observations. More experiments are needed to validate whether the initial difference of the Zeta potential between rutile and anatase is attributed to the synthesis process or not.

**Figure 4:**
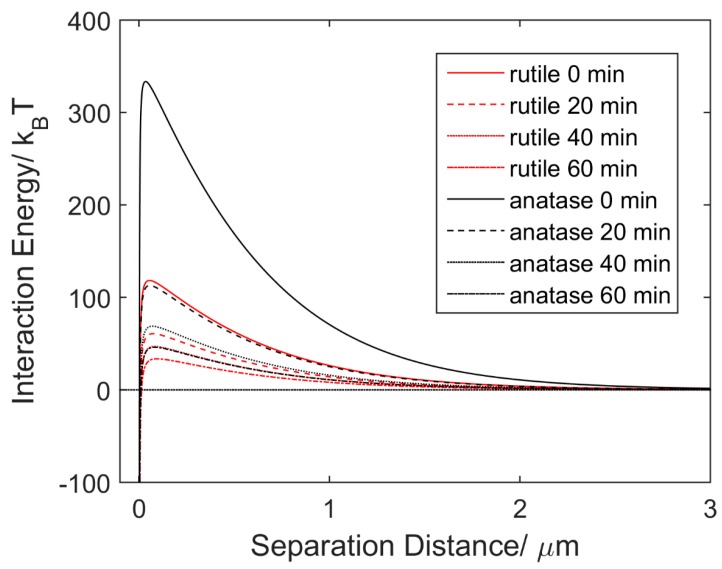
Changes of interaction energy of rutile versus anatase submicron particles in DI water over time under UV irradiation.

The DLVO theoretical model was applied to quantitatively investigate the aggregation behaviors of rutile and anatase particles and the results are shown in Figure 4. The EDL repulsive force is determined by the particle surface charge. The initial Zeta potential of anatase was set to −30 mV in DI water (pH ≈ 6.5) as a reference point [17]. From the difference of measured mobility shown in Figure 3, the initial Zeta potential of rutile was calculated as −18.3 mV based on Equations (1) and (2). After 20, 40 and 60 min of UV irradiation, the Zeta potentials of rutile and anatase were reduced to −13.4, −11.9, −10.2 mV and −17.9, −14.2, −11.8 mV, respectively, according to the decrease of the particle mobility.

As shown in Figure 4, the interaction energy curves illustrate a significant influence of prolonged UV irradiation on energy barriers for rutile and anatase, respectively. Based on Equations (3) and (4), the energy barriers of rutile and anatase were simulated as a function of irradiation time. Compared to the initial energy barrier of 333.5 k_B_T and 118.2 k_B_T, the maximum interaction energy decreased to only 46.1 k_B_T and 33.6 k_B_T for rutile and anatase, respectively, after 1 h of UV irradiation. Such a significant reduction of energy barriers indicates that UV irradiation facilitates the aggregation of rutile and anatase submicron particles. Furthermore, the faster aggregation rate of rutile particles could be a result of its lower energy barrier throughout UV exposure.

## 5. Conclusions

UV light–controlled aggregation of TiO_2_ submicron particles in aqueous suspensions was investigated and it was shown that aggregation of both rutile and anatase particles can be facilitated by UV irradiation. Upon UV exposure, the measured mobility significantly decreased for both rutile and anatase particles, indicating a reduction of the Zeta potential. The reduction of the Zeta potential enhanced the attraction among TiO_2_ particles, and therefore facilitated aggregation. In the experiments, rutile particles exhibited a much faster aggregation rate than anatase particles and rutile particles showed a lower mobility (a more neutral Zeta potential) than anatase particles under UV irradiation. This was concluded to be caused by the lower repulsive interaction which led to the observed faster aggregation rate of rutile particles. It is possible that the faster agglomeration rate of as-prepared rutile might come from its synthesis process. The obvious decrease of the barrier energy simulated through the DLVO model quantitatively confirmed the notable effects of UV light on rutile and anatase particle aggregation. This work contributes to further understanding of the fundamentals of light-controlled micro/nanoparticles in aqueous media and holds considerable promise for environmental remediation.
